# Association between COVID-19 lockdown measures and the incidence of iatrogenic versus spontaneous very preterm births in the Netherlands: a retrospective study

**DOI:** 10.1186/s12884-021-04249-8

**Published:** 2021-11-12

**Authors:** J. Klumper, B. M. Kazemier, J. V. Been, K. W. M. Bloemenkamp, M. A. de Boer, J. J. H. M. Erwich, W. Heidema, F. J. C. M. Klumper, S. W. A. Nij Bijvank, S. G. Oei, M. A. Oudijk, S. Schoenmakers, A. S. van Teeffelen, C. J. M. de Groot

**Affiliations:** 1 Department of Obstetrics and Gynaecology, Amsterdam Reproduction and Development center, Amsterdam UMC, location AMC, Meibergdreef 9, 1105 AZ Amsterdam, The Netherlands; 2 Division of Neonatology, Department of Pediatrics, Erasmus MC-Sophia Children’s Hospital, University Medical Center Rotterdam, Rotterdam, The Netherlands; 3 Department of Obstetrics and Gynaecology, Erasmus MC-Sophia Children’s Hospital, University Medical Center Rotterdam, Rotterdam, The Netherlands; 4 Department of Public Health, Erasmus MC, University Medical Centre Rotterdam, Rotterdam, The Netherlands; 5 Department of Obstetrics, Wilhelmina Children Hospital, University Medical Center Utrecht, Utrecht University, Utrecht, The Netherlands; 6 Department of Obstetrics and Gynaecology, Amsterdam Reproduction and Development center, Amsterdam UMC, location VUmc, Amsterdam, The Netherlands; 7 Department of Obstetrics and Gynaecology, University Medical Center Groningen, University of Groningen, Groningen, The Netherlands; 8 Department of Obstetrics and Gynaecology, Radboud University Medical Center, Nijmegen, The Netherlands; 9 Department of Obstetrics and Gynaecology, Leiden University Medical Center, Leiden, The Netherlands; 10 Department of Obstetrics and Gynaecology, Isala Women’s and Children’s hospital, Zwolle, the Netherlands; 11 Department of Obstetrics and Gynaecology, Máxima Medical Center, Veldhoven, the Netherlands; 12 Eindhoven MedTech Innovation Center (e/MTIC), Eindhoven, the Netherlands; 13 Department of Electrical Engineering, Eindhoven University of Technology, Eindhoven, the Netherlands; 14 Department of Obstetrics and Gynaecology, Grow, School for Oncology and Developmental Biology, Maastricht University Medical Centre (MUMC+), Maastricht, The Netherlands

**Keywords:** COVID-19, Lockdown, Preterm birth, Iatrogenic, Spontaneous

## Abstract

**Background:**

The COVID-19 pandemic led to regional or nationwide lockdowns as part of risk mitigation measurements in many countries worldwide. Recent studies suggest an unexpected and unprecedented decrease in preterm births during the initial COVID-19 lockdowns in the first half of 2020. The objective of the current study was to assess the effects of the two months of the initial national COVID-19 lockdown period on the incidence of very and extremely preterm birth in the Netherlands, stratified by either spontaneous or iatrogenic onset of delivery, in both singleton and multiple pregnancies.

**Methods:**

Retrospective cohort study using data from all 10 perinatal centers in the Netherlands on very and extremely preterm births during the initial COVID-19 lockdown from March 15 to May 15, 2020. Incidences of very and extremely preterm birth were calculated using an estimate of the total number of births in the Netherlands in this period. As reference, we used data from the corresponding calendar period in 2015–2018 from the national perinatal registry (Perined). We differentiated between spontaneous versus iatrogenic onset of delivery and between singleton versus multiple pregnancies.

**Results:**

The incidence of total preterm birth < 32 weeks in singleton pregnancies was 6.1‰ in the study period in 2020 versus 6.5‰ in the corresponding period in 2015–2018. The decrease in preterm births in singletons was solely due to a significant decrease in iatrogenic preterm births, both < 32 weeks (OR 0.71; 95%CI 0.53 to 0.95) and < 28 weeks (OR 0.53; 95%CI 0.29 to 0.97). For multiple pregnancies, an increase in preterm births < 28 weeks was observed (OR 2.43; 95%CI 1.35 to 4.39).

**Conclusion:**

This study shows a decrease in iatrogenic preterm births during the initial COVID-19-related lockdown in the Netherlands in singletons. Future studies should focus on the mechanism of action of lockdown measures and reduction of preterm birth and the effects of perinatal outcome.

## Introduction

The outbreak of the coronavirus disease 2019 (COVID-19) resulting from SARS CoV-2 has a major impact on healthcare worldwide, and was declared an official pandemic by the World Health Organization (WHO) on March 11, 2020 [1]. Strict mitigation measurements and national lockdowns were implemented to prevent spread of infection and diminish its effects. The mitigation measures have affected health care infrastructure and logistics, changing patterns of hospitals contact for all medical conditions [2].

In pregnant women with severe COVID-19, the preterm birth risk appears to be increased [3, 4]. However, at a population level, reports from The Netherlands, Denmark and Ireland indicate an unprecedented decrease in the overall incidence of preterm births during national lockdown periods [5–7].

While the COVID-19 pandemic rages on, threatening the lives of many adults, the finding that preterm birth decreased during the first lockdown warrants our full attention, since preterm birth affects millions of families worldwide each year.

Preterm birth, defined as birth before 37 + 0 weeks of gestation, is a major contributor to perinatal mortality and morbidity, yearly complicating over 15 million pregnancies worldwide [8]. Prevention of preterm birth has been an important global goal for all obstetricians, recognized by the WHO in 2012 in the publication of the report ‘Born too soon: the global action report on preterm birth’. [9] Although some effective prevention strategies have been developed such as progesterone or pessary, they unfortunately have not resulted in an impressive reduction of preterm birth rates. More detailed information on the true effects of lockdown measures in the COVID-19 pandemic on preterm birth can aid in the development of new measures to reduce preterm births.

The previously mentioned studies showed a decrease in preterm birth in COVID-19 lockdown used a relatively small sample size [6, 7], excluded multiple births [5, 6] and could not differentiate between spontaneous or iatrogenic causes of preterm birth [5–7]. To understand the underlying mechanisms leading to preterm birth, a clear distinction in type of onset of preterm delivery is important.

In this study, we collected data on preterm births < 32 weeks of gestation from all ten perinatal centers with level-III Neonatal Intensive Care Facilities in the Netherlands. Our aim was to assess the association between the initial COVID-19 lockdown in the Netherlands and the incidence of preterm birth < 32 weeks of gestation, taking onset of delivery and composition of the pregnancy (singleton versus multiple) into account.

## Methods

### Setting

The first national COVID-19 mitigation measures in the Netherlands were introduced on March 9, 2020. These measures were intensified on March 15, 2020: childcare facilities and non-essential services involving physical contact were closed and social distancing was introduced (1.5 m rule). Most mitigation measures were gradually phased out in May and June 2020. With the opening of restaurants and public facilities (e.g. libraries, theaters, museums) on May 15, 2020, the first episode of most rigorous COVID-19 mitigation measures ended. For this study, we selected the period from March 15 up to and including May 15, 2020.

### Data from 2020

In the Netherlands, pregnant women with signs of preterm labor < 32 weeks of gestation or with a high risk of requiring iatrogenic preterm birth < 32 weeks are transferred to one of the ten perinatal centers with a specialized neonatal intensive care unit (NICU) if possible [10]. Therefore, majority of all births from 24^+ 0^ weeks to 31^+ 6^ weeks – i.e. very preterm and extremely preterm - take place in one of these centers. Dutch national guidelines advise against active management of neonates born at gestational ages less than 24^+ 0^ weeks [11].

#### The nominator

Data on all births from March 15 to May 15, 2020 with a gestational age between 24^+ 0^ weeks to 31^+ 6^ weeks, were provided by each perinatal centers as derived from local medical files. Collected items were: date of birth, gestational age at birth, type of onset of labor (spontaneous or iatrogenic), pregnancy composition (singleton or multiple), mode of delivery (vaginal or cesarean), child sex and birth weight. Data on maternal age, parity, previous obstetric history, BMI, pre-existing chronic disease, socio-economic status, ethnicity or other risk factors for preterm birth were not collected given the exploratory nature of the study.

#### The denominator

The total number of births in the study period in 2020 was obtained through the National Institute for Public Health and the Environment (RIVM) as extracted from Praeventis. In the Netherlands, all neonates are screened for a number of diseases after 72 h of birth using a dried blood spot card as part of our national screening program. Data about this screening and the provided limited maternal and neonatal characteristics such as gestational birth are collected in a national database called Praeventis [12, 13]. Preterm neonates are screened without delay [14]. Multiple gestations were identified by selecting records with identical surnames, birth dates and postal codes, thereby deriving the total number of pregnancies in the study period in 2020.

### Data from 2015 to 2018; reference group

Since Praeventis contains no information on start of delivery (spontaneous vs iatrogenic), we were unable to use this database for our reference group. We therefore obtained data on preterm births between 24^+ 0^ weeks to 31^+ 6^ weeks in the corresponding calendar period (March 15 to May 15) in 2015 to 2018 from The Netherlands national perinatal registry, Perined [15]. The Perined registry consists of population-based data containing information on pregnancy, delivery and neonatal outcomes. The Perined database is a validated linkage of three different registries: the midwifery registry, the obstetrics registry and the neonatology registry. Perined data are typically published 1–2 years after initial registration of pregnancies and births, and as such data from 2019 and 2020 were not available at the time of this study, hence the use of Praeventis. Despite national guidelines, a minority of neonates born < 32 weeks are born outside one of the ten perinatal centers with a specialized NICU. In the reference nominator, we therefore only included very preterm and extremely preterm births from the 10 perinatal centers as was collected in the year 2020.

Since we were only able to collect data about live births during the lockdown period in 2020, and the Praeventis database only collects data on neonates having undergone neonatal screening, we excluded all cases with ante-partum and intra-partum fetal deaths, both in 2020 and in the reference years 2015–2018 .

The Perined registry from 2015 to 2018 covers around 96–98% of all births [15], whereas the Praeventis database covers around 99% of all newborns [16]. By subtracting the number of births in Perined by the numbers of births in Praeventis (years 2015–2019), we were able to identify 735 singletons and 100 multiples that were not registered in Perined. Therefore, these numbers were added to the denominator of the reference years 2015–2019.

### Outcome

Our primary outcome was preterm birth < 32 weeks (very preterm) and < 28 weeks (extremely preterm) in singleton and multiple pregnancies. Gestational age was predominantly based on crown rump length measurement in early pregnancy. In addition, we stratified the analysis by singletons and multiples and by type of onset of preterm birth (spontaneous vs iatrogenic). Preterm births were considered ‘spontaneous’ if labor started with spontaneous preterm contractions and/or preterm prelabor rupture of membranes (PPROM). Preterm births after elective cesarean section or after induction of labor were considered to have an ‘iatrogenic’ onset.

### Statistical analyses

Baseline characteristics of the 2020 preterm cohort and the 2015–2018 preterm cohort were compared using Student’s t-test and chi-squared test or Fisher’s exact tests where appropriate. Proportions of preterm birth for the subgroups were calculated and compared with chi-square test. Logistic regression was used to express an increased or decreased risk of preterm birth in the cohort of 2020 using unadjusted Odds Ratios (OR) with 95% confidence intervals (95%CI). All analyses were performed using SAS Statistics, version 9.4.

### Ethics approvals

The Medical Research Ethics Committee at the Amsterdam University Medical Centers, location VUmc, approved this study (approval number 2020.480).

Second, The National Institute for Public Health and the Environment approved the study protocol for using Praeventis data. Third, Perined approved the use of their data (approval number 20.14) for the years 2015–2018.

### Patient involvement

None.

## Results

From March 15 to May 15, 2020, Praeventis registered 27,351 live born children, of which 847 were part of multiple pregnancies. Accordingly, we conclude that there were 26,504 singleton births, and 420 multiple births.

In this cohort, we observed 200 live births with a gestational age < 32 weeks (gestational age between 24^+ 0^ weeks to 31^+ 6^ weeks). Of these births, 163 were singleton and 37 were multiple births. Two twin pregnancies had undergone fetal foeticide because of congenital anomalies before 24 weeks, they were still categorized as twins.

Baseline comparison between the 2020 cohort of preterm births < 32 weeks in the corresponding calendar period and the 2015–2018 cohort showed no significant differences, Table 1.

**Table 1:** Baseline characteristics of both cohorts

Characteristics	Cohort 2020	Cohort 2015–2018	*p*-value
	GA < 32 weeks	GA < 32 weeks	
	*N* = 200 births	*N* = 829 births	
Gestation age (GA) in days, mean (SD)	200.1 (16.6)	202.6 (14.7)	0.051
Multiple gestation, no (%)	37 (18.5)	138 (16.6)	0.53^a^
Mode of delivery^b^			
Singletons			0.63
Vaginal, no (%)	80 (49.0)	314 (47.3)	
Cesarean section, no (%)	82 (51.0)	350 (52.7)	
Multiple gestation			0.73
Vaginal, no (%)	21 (56.8)	69 (53.5)	
Cesarean section, no (%)	16 (43.2)	60 (46.5)	
Sex			0.79
Male, no (%)	125 (53.0)	520 (54.6)	
Female, no (%)	108 (45.8)	432 (45.4)	
Birth weight in grams, mean (SD) ^c^	1189 (410)	1214 (391)	0.62
Birthweight <5th percentile (Hoftiezer curve), no (%)^d^	46 (19.3)	219 (23.0)	0.22

*GA* gestation age, *SD* standard deviation

^a^ Pearson Uncorrected Chi-square

^b^ missing *N* = 32, some multiple pregnancies delivered one child vaginally and the other by caesarean

^c^ missing *N* = 1

^d^ Calculated over the number of children with available birth weight *N* = 238 in 2020, *N* = 951 in 2015–2018

Table 2 shows the number of preterm births (singletons and multiples) stratified per year. It shows that the incidence of preterm birth was fairly stable over the years in the same calendar period with the exception of a lower incidence in the year 2020.

**Table 2:** Total preterm births in the ten perinatal centers (singleton + multiples), per year

All births (singleton + multiples)	Cohort 2015	Cohort 2016	Cohort 2017	Cohort 2018	Cohort 2020
	*N* = 26,437	*N* = 27,504	N = 26,780	N = 26,605	N = 26,924
Total PTB < 32 weeks, no (‰)	234 (8.9)	241 (8.7)	240 (9.0)	227 (8.5)	200 (7.4)
Spontaneous PTB < 32 weeks, no (‰)	128 (4.8)	141 (5.1)	144 (5.4)	120 (4.5)	141 (5.2)
Iatrogenic PTB < 32 weeks, no (‰)	106 (4.0)	100 (3.6)	96 (3.6)	107 (4.0)	59 (2.2)
Total PTB < 28 weeks, no (‰)	65 (2.5)	64 (2.3)	81 (3.0)	75 (2.8)	76 (2.8)
Spontaneous PTB < 28 weeks, no (‰)	39 (1.5)	40 (1.5)	55 (2.1)	45 (1.7)	63 (2.3)
Iatrogenic PTB < 28 weeks, no (‰)	26 (1.0)	24 (0.9)	26 (1.0)	30 (1.1)	13 (0.5)

*PTB* preterm birth

### Singletons

**Fig. 1:**
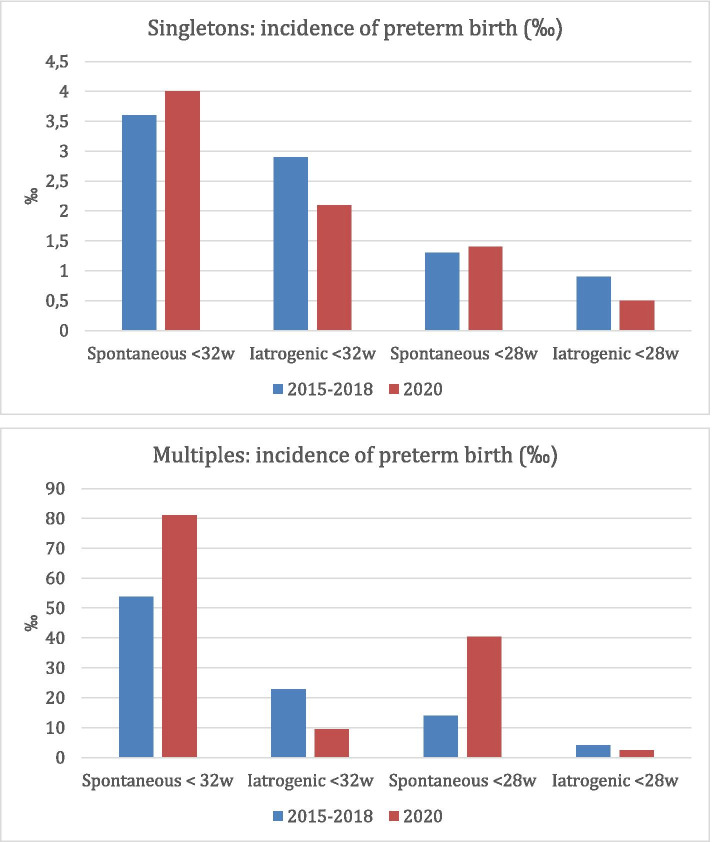
Incidences of preterm birth stratified by singletons and multiples

The incidence of total preterm birth < 32 weeks in singleton pregnancies was 6.1‰ in the study period in 2020 versus 6.5‰ in the corresponding period in 2015–2018 (OR 0.94; 95%CI 0.79 to 1.12, Table 3, Fig. 1). The decrease in preterm births in singletons was solely due to a decrease in the iatrogenic preterm births, both < 32 weeks (OR 0.71; 95%CI 0.53 to 0.95) and < 28 weeks (OR 0.53; 95%CI 0.29 to 0.97, Table 3). The incidence of spontaneous preterm birth in 2020 compared to 2015–2018 was not significantly different, both in the < 32 weeks group (OR 1.13; 95%CI 0.91 to 1.40) and in the < 28 weeks group (OR 1.31; 95%CI 0.94 to 1.83, Table 3).

**Table 3:** Incidences preterm birth stratified by singletons and multiples

	Cohort 2020		Cohort 2015–2018			
	N	‰	N	‰	OR	95%CI
**Singletons**	**N = 26,504**		***N*** **= 106,468**			
Total PTB < 32 weeks	162	6.1	691	6.5	0.94	0.79–1.12
Spontaneous PTB < 32 weeks	107	4.0	380	3.6	1.13	0.91–1.40
Iatrogenic PTB < 32 weeks	55	2.1	311	2.9	**0.71**	**0.53–0.95**
Total PTB < 28 weeks	58	2.2	232	2.2	1.01	0.75–1.34
Spontaneous PTB < 28 weeks	46	1.7	141	1.3	1.31	0.94–1.83
Iatrogenic PTB < 28 weeks	12	0.5	91	0.9	**0.53**	**0.29–0.97**
**Multiples**	***N*** **= 420**		***N*** **= 1713**			
Total PTB < 32 weeks	38	90.5	131	76.5	1.20	0.82–1.75
Spontaneous PTB < 32 weeks	34	81.0	92	53.7	1.55	1.03–2.36
Iatrogenic PTB < 32 weeks	4	9.5	39	22.8	0.41	0.15–1.16
Total PTB < 28 weeks	18	42.9	31	18.1	**2.43**	**1.35–4.39**
Spontaneous PTB < 28 weeks	17	40.5	24	14.0	**2.97**	**1.58–5.58**
Iatrogenic PTB < 28 weeks	1	2.4	7	4.1	0.58	0.07–4.74

*OR* odds ratio, *CI* confidence interval, *PTB* preterm birth

### Multiples

The incidence of total preterm birth < 32 weeks in multiple pregnancies was 90.5‰ in the study period in 2020, versus 76.5‰ in the corresponding period in 2015–2018 (OR 1.20; 95%CI 0.82 to 1.75, Table 3, Fig. 1). Other than in singletons, the incidence of iatrogenic preterm birth in 2020 compared to 2015–2018 was not statistically significantly different for multiples, both in the < 32 weeks group (OR 0.41; 95%CI 0.15 to 1.16) and in the < 28 weeks group (OR 0.58; 95%CI 0.07 to 4.74, Table 3). We observed a statistically significant increase in the preterm births < 28 weeks (OR 2.58; 95%CI 1.38 to 4.79), solely due to an increase in births with spontaneous onset (OR 2.97; 95%CI 1.58 to 5.58, Table 3).

## Discussion

### Main findings

Our study reports on both iatrogenic and spontaneous preterm birth changes stratified by singleton and multiple pregnancies. We found a decrease in very and extremely preterm births with iatrogenic onset in singletons during the COVID-19 related lockdown in the Netherlands between March 15 and May 15 in 2020 compared to the corresponding calendar period in 2015–2018. For multiple pregnancies, an increase in extremely preterm births (< 28 weeks) was observed in the study period, solely due to an increase in births with spontaneous onset.

### Strengths & limitations

We present a study assessing the association between COVID-19 lockdown and preterm births which was able to stratify between spontaneous or iatrogenic onset of preterm birth. As both types have a different etiology, distinction between these types is essential for correct interpretation of the effects, especially since we showed that the observed decrease is solely attributable to iatrogenic preterm births. Second, we were able to stratify between singleton and multiple pregnancies, although the total number of multiples were low.

We acknowledge the following limitations:

1. We used manually collected data from 10 perinatal centers, and used two different databases to compare the incidence of preterm during the COVID-19 lockdown period with previous years. The national databases used well-defined definitions for spontaneous or iatrogenic preterm birth, however due to the manual approach, differences in interpretation and registration might have occurred. Furthermore, the Praeventis database covers a higher percentage of births compared to the Perined database (99% versus 96%). We attempted to account for this issue by adding these births to the denominator in the reference group.
2. We were only able to assess births between 24^+ 0^ and 31^+ 6^ weeks of gestation. If lockdown measures indeed resulted in delay of delivery, it is possible that less women delivered with a gestational age less than 24 weeks, thereby influencing the incidence of PTB, especially < 28 weeks of gestation or more women between 32 and 37 weeks. The 2020 Perined data will shed light on this issue.
3. No data from 2020 on perinatal deaths could be collected (nor ante-partum fetal nor intra-partum fetal death), as many of these deliveries occur outside perinatal centers and the national database Perined is not yet available for 2020. In addition, early neonatal deaths were not collected since Praeventis screening is after 72 h. In addition, we could not collect data on maternal characteristics and neonatal outcomes.
4. This study collected data from a limited time period (March–May 2020). We were therefore only able to compare the number of preterm births to the number of women who delivered in that period. A more precise comparison would have been if we were able to take into account all pregnant women in that period, including the women that remained pregnant / whom did not deliver preterm, and construct a time-to-event analysis. Unfortunately, due to the nature of this study this was not possible.

A growing number of studies have revealed a decrease in preterm births during national COVID-19 lockdowns. This study supports evidence from previous studies around the globe showing a decrease in preterm births [5–7, 17–19]. Using the nationwide Praeventis data on neonatal dried blood sampling, Been et al. showed a reduction in the incidence of preterm birth (OR 0.77; 95%CI 0.66–0.91) that was fairly consistent across all preterm gestational age strata in the Netherlands [5]. In a nationwide study from Denmark, data from 31,180 singleton births between March 12 and April 14 in the years 2015–2020 were analyzed. The authors identified a large reduction in extremely preterm births < 28 weeks (OR 0.09; 95%CI 0.01–0.40), although based on only one case of extremely preterm birth [6]. Two studies (from Israel and Italy) also found a decrease in total preterm births [17, 19], but 2 other studies (from Sweden, Ireland) found no change in preterm births during national COVID-19 lockdowns [20, 21]. However, none of these studies were unable to stratify between iatrogenic and spontaneous preterm birth. A single center study from the USA was able to differentiate between spontaneous and iatrogenic preterm birth and found a reduction in iatrogenic preterm birth < 34 week (OR 0.40; 95%CI 0.21–0.75) in concordance to our results [18]. In contrast to earlier findings, authors in Nepal observed an increase of overall preterm birth < 37 weeks, with an adjusted risk ratio during lockdown versus before lockdown of 1.30 (95%CI 1.20–1.40) [22].

### Interpretation

Several theories have been put forward to explain the decrease in preterm birth during the COVID-19 lockdowns observed in various settings. Possible affected risk factors include a reduction in asymptomatic maternal infections due to hygiene measures, less physical activity and minimal direct social interaction due to home confinement, less work-related stress and reductions in air pollution. Alteration in these factors mainly influence the risk of spontaneous preterm birth [23]. However, in our opinion, these theories are less explanatory for the decrease we found in iatrogenic preterm birth.

There are several possible explanations for a decrease in iatrogenic preterm births.

First, a decrease in the incidence of preeclampsia, fetal growth restriction or placental abruption would probably lead to a reduction in iatrogenic preterm births. During the initial COVID-19 lockdown pregnant women experienced less physical activity due to home confinement, and could take more rest during the day. Lockdown measures may have influenced the severity of placental obstetrics syndromes such as preeclampsia and fetal growth restriction, thereby reducing the need for early intervention by induction of labor or planned cesarean section. However, there is currently not enough evidence affirming rest is helpful in preventing pre-eclampsia and its complications [24]. Based on the results of this study, advising pregnant woman to take more rest or advise other life-style changes in non-lockdown periods would be too premature.

Second, a more likely explanation could be found in the patterns of hospital visits and admission for pregnant women at risk of iatrogenic preterm birth. Women might have been reluctant to visit the hospital for fear of contracting infection in the hospital [25], or not wanting to weigh down the health care burden. Previous studies showed that pregnant women were substantially psychologically affected during the pandemic and consequently changed their care seeking behavior [26]. Additionally, general practitioners and midwives might have experienced a threshold to refer pregnant women to the hospital [27].

Third, during the COVID-19 lockdown, consultations with the midwives and antenatal outpatient clinic visits were reduced. Physical appointments were replaced by telephone appointments, without blood pressure measurement or ultrasound examination. Overall increases in telemedicine during COVID-19 quarantine up to 68% have been reported, which could have impacted quality of care [28]. Possibly, hypertension or growth restriction was diagnosed later than normal, shifting to births at a slightly higher gestational age.

Unfortunately, we have no data on maternal morbidity and adverse perinatal outcome during the studied period. Stowe et al. found no evidence of an increase in stillbirths regionally or nationally during the COVID-19 pandemic in England when compared with the same months in the previous year [29]. We cannot determine whether the observed reduction in our study occurred at the expense of increased maternal and/or fetal morbidity and mortality, such as stillbirths. This is an important knowledge gap as downscaling regular obstetric care might not be without consequences. Alternatively, reducing the number of antenatal visits could be considered if future studies conclude that more frequent antenatal visits lead to unnecessary interventions without improving the outcome for mother and child.

As Perined data will become available for 2020, we plan to validate our findings by undertaking a methodologically much more robust analysis of national data from a single data source, which will allow taking into account underlying temporal trends, appropriate adjustment for confounding, and exploration of subgroup-specific effects. Besides smaller initiatives, an international network has been set up to further investigate lockdown effects on perinatal outcome: the iPOP study [30]. Hopefully these initiatives will aid in determining unrecognized or preventable factors related to preterm birth with the ultimate goal to finally successfully decrease the incidence of preterm birth in the future.

## Conclusion

Our study confirms findings from a previous quasi-experimental study that the incidence of preterm birth was reduced following lockdown measures in the Netherlands. We provide essential novel information by indicating that the observed reduction was mainly due to iatrogenic and not spontaneous preterm births. Future studies should direct their focus on why the number of iatrogenic births were decreased and at what possible costs or benefits (e.g. maternal morbidity, perinatal mortality, less intervention).

## Data Availability

The datasets used and/or analysed during the current study are available from the corresponding author on reasonable request.

## Abbreviations

COVID-19: Coronavirus disease 2019
PTB: Preterm birth
PPROM: Preterm prelabor rupture of membranes
